# The relational shift in urban ecology: From place and structures to multiple modes of coproduction for positive urban futures

**DOI:** 10.1007/s13280-024-02001-y

**Published:** 2024-04-20

**Authors:** Steward T. A. Pickett, AbdouMaliq T. Simone, Pippin Anderson, Ayyoob Sharifi, Aliyu Barau, Fushcia-Ann Hoover, Daniel L. Childers, Timon McPhearson, Tischa A. Muñoz-Erickson, Chantal Pacteau, Morgan Grove, Niki Frantzeskaki, Harini Nagendra, Joshua Ginsberg

**Affiliations:** 1 Cary Institute of Ecosystem Studies, Millbrook, NY 12545 USA; 2 Urban Institute, University of Sheffield, Sheffield, UK; 3 Department of Environmental and Geographical Science, University of Cape Town, Rondebosch, Private Bag x3, Cape Town, 7701 South Africa; 4 Hiroshima University, 1-5-1 Kagamiyama, Higashi-Hiroshima, Hiroshima 739-8529 Japan; 5 Department of Urban and Regional Planning, Bayero University Kano, PMB 3011, Kano, Nigeria; 6 Department of Geography and Earth Sciences, University of North Carolina, Charlotte, 9201 University City Blvd, Charlotte, NC 28223 USA; 7 School of Sustainability, WCPH 442, Arizona State University, POB 877904, Tempe, AZ 85287-7904 USA; 8 The New School, 79 Fifth Avenue, 16th Fl., New York, NY 10003 USA; 9 Stockholm Resilience Centre, Stockholm University, Stockholm, Sweden; 10 International Institute of Tropical Forestry, USDA Forest Service, 1201 Calle Ceiba, Jardín Botánico Sur, Río Piedras, PR 00926 USA; 11 Institut d’Écologie et des Sciences de l’Environnement de Paris, Campus Pierre et Marie Curie 4, place Jussieu, 75005 Paris, France; 12 Baltimore Field Station, USDA Forest Service, 5523 Research Park Drive, Suite 350, Baltimore, MD 21218 USA; 13 Utrecht University, Vening Meinesz Building A, Princetonlaan 8a, 3584 CB Utrecht, The Netherlands; 14 Centre for Climate Change and Sustainability, Azim Premji University, Burugunte Village, Bikkanahalli Main Road, Sarjapura, Bangalore, 562125 India; 15 Beyond Inhabitation Lab, Polytechnic University of Turin, Turin, Italy

**Keywords:** Coproduction, Equity, Global change, Human ecosystem, Social-ecological-technological system, Urban ecology

## Abstract

This perspective emerged from ongoing dialogue among ecologists initiated by a virtual workshop in 2021. A transdisciplinary group of researchers and practitioners conclude that urban ecology as a science can better contribute to positive futures by focusing on relationships, rather than prioritizing urban structures. Insights from other relational disciplines, such as political ecology, governance, urban design, and conservation also contribute. Relationality is especially powerful given the need to rapidly adapt to the changing social and biophysical drivers of global urban systems. These unprecedented dynamics are better understood through a relational lens than traditional structural questions. We use three kinds of coproduction—of the social-ecological world, of science, and of actionable knowledge—to identify key processes of coproduction within urban places. Connectivity is crucial to relational urban ecology. Eight themes emerge from the joint explorations of the paper and point toward social action for improving life and environment in urban futures.

## Introduction: A multifaceted perspective

This paper presents a group perspective that emerged from an international, multidisciplinary process to position urban ecology as a science to better contribute toward positive urban futures. We hope this positioning helps those beyond ecology see points of collaboration. The goal of this paper is to summarize the insights that the authors arrived at during and since the conference about a relational shift in urban ecology. The relational shift suggests that relationships and their implications, rather than city or urban structure, are now a primary focus of the field. Relational urban ecology is defined as a subset of the science of urban ecology that focuses first on relationships among the social and ecological components of urban ecosystems. Focus on structure, which has historically been paramount in urban ecology, is placed secondary to a focus on relationships. Because urban ecology is a subset of ecology its relationships involve organisms and the energetic, material, and informational transformations they engage in along with the transformations due to social power differentials, economies, social identity, institutional arrangements, human demography, and senses of place, among others. While we acknowledge that political ecology has contributed much to the understanding (and critiquing) urban places (Jaret 1983; Heynen et al. 2006; Gandy 2015, 2022; Rademacher 2015) we focus here on urban ecological science as our shared expertise.

Defining urban relational ecology as we have done just above, exercises one of three major ways the term “ecology” can be used (Pickett and Cadenasso 2002). The relational ecology definition employs a technical view that emerged from within the science of ecology itself (e.g., Weathers et al. 2021). But beyond a technical definition, ecology may also refer to a specific model of some place or system. The energy flow simulation of an ecosystem, or the biodiversity structure of a forest are examples. It is possible that other disciplines may employ models which are essentially ecological in structure, that is, composed of entities that interact and transform matter, energy, and information. Such models may be metabolic (Inostroza 2014). The final way to use the term ecology is as a metaphor, that is an image or a cultural marker. Such metaphors appear in urban design and planning (McGrath 2013). In this paper we emphasize the technical or modeling uses, although we have occasion to acknowledge the role of metaphor in relational work.

The 14 authors of this paper are a subset of the 35 participants of the larger workshop that identified the relational shift. The collective primarily consisted of self-identified urban ecologists and sustainability scientists, but the group also represented science policy, urban design, engineering, nature-based solutions, environmental justice, governance, and climate, among others. The roster of authors includes those who reside or work in the Global South. The conference took place during the COVID-19 lockdowns of 2021, mandating a virtual meeting. The schedule accommodated a broad range of time zones. The authors represent a broad geography, with a bounding polygon marked by Japan, India, Australia, South Africa, Sweden, the western US, and Colombia. In between were representatives from China, Nigeria, Germany, the UK, France, and the Netherlands.

The group’s deliberations identified two large ideas this paper will explore.

- Eight key issues or themes characterize the empirical, conceptual, and practical status the field of urban ecology, identifying the gaps it can better fill, and highlighting interdisciplinary insights it can exploit;
- Key kinds of coproduction—of science, of the urban subject of study, and of knowledge to support equitable action—can organize the frontier work remaining. Notably, only the knowledge outcome is usually labeled coproduction at present.

The themes are only enumerated here, but will be unpacked and referenced later in the paper. The themes have many sources, some from well outside the field of urban ecology. The novelty is not in the themes themselves, which in any event we do not claim as ours, but in combining them to distill their implications for the science of urban ecology.

1. Urban isn't just "the city," (Lefebvre 2003; Sassen 2010; Brenner 2014b; Gandy 2014), although the term "city" may be used as a short hand label.
2. Urban systems can be remarkably dynamic, reflecting both internal and external drivers (e.g., Shane 2005; Zhou et al. 2021a, b).
3. The first two themes suggest novel ways of shaping how urban ecological science can contribute to thinking about, planning, and managing "cities"
4. Urban relationships extend over time and space, with vast technological and other kinds of connectivity (Liu et al. 2007; Brondizio et al. 2016).
5. Power differentials and relationships are key to governance of urban ecology, and to the pursuit of equity and justice (Anguelovski 2013; Heynen et al. 2018; Anguelovski and Connolly 2021).
6. Thinking beyond the Western people vs nature divide, or beyond models from the Global North, promotes a broader view of relevant evidence and effective environmental knowledge (McHale et al. 2015). See also the contribution by Andersson et al. (2024) to this special feature.
7. Urban ecology is not a universalizing discipline, but rather a field in which the positionality of scientists, and the contexts of the places studied is highly significant to success and relevance.
8. Urban ecology has great opportunity to advance equity and justice across the unequal power networks of urban places. (See also the contribution by Grove et al. (2024) to this special feature.)

The authors jointly identified these themes to reflect the increasingly relational approach to urban ecology. These themes emerge from two major kinds of transitions (cf. Frantzeskaki et al. 2024 in the special feature). One is the field’s increasing interaction with other disciplines (Section II). As we shall expand below, these influential fields include the social sciences of political ecology with its emphasis on power differentials, governance with its broad understanding of modes of decision making, the practice of urban design, and the science of conservation. The list is diverse, but intentionally not comprehensive.

The other source of the themes is the evolving conditions driving rapid change in the urban world (Section III). These include global climate change with its consequences, digital and other technological changes, the growing regional and global connectivity of people, materials, energy, information, and capital, and finally, the growing calls for justice and equity. Contexts are detailed and referenced below.

Once those tasks are accomplished, the paper returns to one of the group’s big ideas, the different modes of coproduction (Section IV) that can support the shift of urban ecology toward relationality. Some of the specific social-ecological-technological processes by which the urban itself is coproduced are exemplified in Section V. These stretch urban ecology well beyond its biological roots. Section VI then links the big idea of connectivity with key mechanisms of urban system coproduction via the continuum of urbanity concept. Section VII unpacks the themes that the group elicited over its two years of virtual dialogue, which included regular exchange of essays, outlines, and draft texts. The paper concludes (Section VIII) with a brief exploration of from relationality to action.

## Insights on relationality from other disciplines

What does relationality mean? We first take a look at how relationality might be described within ecology, and then seek insights from some other disciplines that take a relational perspective.

We expand the brief definition of relational ecology offered above. The relational shift can be taken to prioritize interaction as the principal stimulus for ecological questions concerning urban systems. This contrasts with the tradition of starting ecological research with focus on structure. A structural approach guided the earlier history of ecology in general, and of urban ecology as well (Collins et al. 2000; McDonnell 2011; Solecki et al. 2013; Wentz et al. 2018). The structural focus was often concerned with issues such as: What are the ecological boundaries of urban places? What are their material characteristics? What are the best ways to characterize urban form and extent in their surrounding landscapes (Forman 2014; Wentz et al. 2018)? Of course, scientific knowledge must be based on jointly understanding the interaction–structure nexus, but urban ecology is currently increasing its relational emphasis to rebalance the dialog that had formerly been biased toward structure (Palazzo and Steiner 2011; McPhearson et al. 2016). To a large extent, relational urban ecology shifts focus from the quantification and delimitation of urban entities, to the understanding of the quality of urban places and the human and other-than-human interactions they exhibit (Marcotullio and Solecki 2013).

### An ecology poised for openness

Two characteristics of ecology suggest that it is open to the insights from other disciplines. First, even classical ecology has been well connected with other sciences that help understand the phenomena it studies. Soil science, climatology, geology, hydrology, and crop science have played this role since the early days of ecology. However, more recently, openness to the multiple modes of evolution, and genetics have helped develop ecological science. Human demography and epidemiology have likewise, especially in the last 15 years, stimulated understanding ecological interactions (LaDeau and Han 2016).

Critical reflection within ecological science has contributed to transformations (sensu Frantzeskaki et al. 2024) that support the relationality explored in this paper. First, ecology has matured sufficiently that the mid-twentieth century defensiveness concerning intellectual boundaries has abated. Ecology now commonly operates with porous boundaries (e.g., Weathers et al. 2021). Furthermore, the background assumptions of the discipline—its paradigm—now recognize roles of disturbance, non-deterministic causality, openness of systems to information and materials from outside their recognized boundaries, and entanglement with human presence, artifacts, and legacies (Fiedler et al. 1997; Pulliam and Johnson 2002; Simberloff 2014). This paradigm shift also admits to a breadth of data sources and ways of knowing that can be used to understand and manage ecological systems (Andersson et al. 2024). Narrow positivist philosophy with its emphasis on falsificationism and elevation of quantitative analysis no longer describe the range of methodologies available to ecology (Pickett et al. 2007; Andersson et al. 2024, this special feature). A later section explores the porosity of science to social context (Jasanoff 2004; Longo et al. 2021), and points to Indigenous and local knowledge as legitimate sources for ecology (Zurba et al. 2019; Tengö et al. 2021). The two kinds of openness just described should welcome deeper connections with other disciplines.

### Broadening the connections with other disciplines

With the growth of environmental sciences after Earth Day in 1970, understanding human demography, behavior, economics, and technology have become closer allies in ecological understanding. These contacts have created porosity and creative instability of the intellectual boundaries of urban ecology (Young 2009). Consequently, adjacent relational fields have led, paralleled, or reinforced the refinement and expansion of urban ecology toward relationality. These disciplines are deeply concerned with power, politics, and contestation, and do not assume disinterest, unfettered rationality, and uniform value sets. A shortcoming of the conceptual transformations of ecology in the past is that they were considered to operate in a neutral, apolitical arena (Shapin 2010; Harding 2015). This and many other limitations on ecology have been exposed through interactions with several already relational disciplines. Here are a four examples.

#### Political ecology

Political ecology is a relational field of particular relevance (Robbins 2004; Rademacher 2015; Rademacher and Sivaramakrishnan 2017). It may be said to examine the “institutional arrangements that govern relationships between knowledge and power, science and society, and state and citizens” (Wyborn et al. 2019:319). Campbell and Gabriel (2016:253) critique studies of human-environmental relations for not “sufficiently problematizing the effects of power within these processes.” They go on to point to the “thick relational networks” (p 253) in which urban populations are embedded, requiring greater engagement with critical social theory, of which political ecology is often a representative. An example of questions raised by this approach is, “Whose resilience, whose city?” (Vale 2014).

The urban focus of political ecology is well developed (e.g., Rademacher 2015). Political ecology has a great deal to do with who is exposed to the most impactful global climate changes (Hamstead et al. 2021) and associated disasters (Parthasarathy 2018) and who can avoid them (Lamb and Vale 2023). Clearly, local political filters determine what communities and people benefit from environmental interventions (Douglas 2014). One insight is that traditional power centers are not the only concerns in political ecology (Campbell and Gabriel 2016), which pays attention to marginalized or oppressed populations. Acheampong (2020) suggests that political ecology actually requires greater attention in our field to the unequal power relations to ecosystem services. Indeed, power relations impinge on the kind of research ecology must do in the wake of disaster (Gibson et al. 2021).

#### Governance and governmentality

The close linkage between power and politics (Ahlborg and Nightingale 2018) take the paper next to governance. Ahlborg and Nightingale (2018) emphasize that power is “relational and productive,” rather than an abstract resource that can be held in isolation. This is an important insight for urban ecology. Governance is the complex set of interactions by which power is created, allocated, and enacted. It is much more than formal government, but rather addresses institutions that range from households to state-spanning arrangements (Finewood et al. 2019; Delaroche et al. 2023). It constitutes the whole of power and decision making processes that result in the allocation and use of resources in social-ecological systems (Bai et al. 2010; Muñoz‐Erickson et al. 2016). Governance must thus be considered one of the drivers of urban social-ecological systems dynamics. It operates at various scales and deploys power in multiple ways (Delaroche et al. 2023). According to Muñoz-Erickson et al. (2016), governance consists of the operative network structure and change, the dynamics of knowledge and power, and the outcomes in space and territories. Networks are not fixed over time, and hence their function and the distribution of power likewise can shift through time (e.g., Romolini et al. 2019). An example is how the governance of green stormwater infrastructure differs based on the regulatory versus community-focused motivations for projects in a particular city (Solins et al. 2023).

#### Urban design as a relational practice

The emergence of urban design in the first decades after World War II has been an additional stimulus to relational thinking in ecology. Early conferences on urban design were held in the 1950s in Europe (Shane 2011). The 1956 conference at the Harvard University Graduate School of Design is recognized as a milestone introducing urban design thinking into America. These conferences mark a shift from the coarse scale of urban planning, de-emphasizing the traditional focus on cities as fixed and monumental. Urban design, in contrast, could be considered to focus more on dynamism of sites and their use, and the relationships of public and private spaces to social effects (Oswald and Baccini 2003). Design ideally emerges from the desires of diverse urban actors, not just those of settler-colonial, social, or corporate elites (Hester 2006). Ecology may profit from seeing how urban design increasingly embraces Indigenous place making (Nejad et al. 2020) or the place making of marginalized groups (Hood and Tada 2020). McGrath et al. (2023) include indigenous perspectives and needs in design in integrating village-level design with watershed management, agriculture, and cultural processes in the rapidly changing Chiang Mai region of Thailand. Calls for incorporating indigenous perspectives in urban design are growing apace (Hibbard 2022).

Urban design could also be a platform for experimentation from which both social and environmental lessons can be learned (Felson et al. 2013). Finally, urban design became increasingly important as core metropolitan cities began to diffuse across broader and broader territories, becoming polycentric and fragmented (Graham and Marvin 2001). At the same time, designers became increasingly motivated by preserving or restoring ecological functions in urban places, and in promoting sustainability (Johnson and Hill 2002; Palazzo and Steiner 2011). The adaptive cycle of resilience (Gunderson and Holling 2002) provides architects McGrath and Lei (2021) with a framework for emphasizing relationality of human engagement in the self-organization of cities. Relationality acts via myth, meaning, rules, and norms about the allocation of resources and labor that drive urban change. Admittedly, formal urban design governs far less than the totality of urban change, given that global cities are increasingly receiving large populations that self-build or occupy unplanned and poorly served districts (see subsection below on Making Do, also Fig. 7 in Grove et al. 2024, this special feature).

#### Conservation biology

Conservation of biodiversity and natural amenities began as top-down, literal or quasi-imperial projects. In the USA, the national parks system was formally established in 1916 to preserve presumed vignettes of the continent before European colonization. The first U.S. national park had been declared in 1876 and was administered by the army. Indigenous residents had been removed by force or genocide from Yellowstone National Park. Across the Atlantic, in 1904, South Africa inaugurated its signature Kruger National Park, which ultimately grew to conserve a habitat complex of 19,485 km^2^, supporting charismatic megafauna. Indigenous peoples were removed from Kruger. Park employees were sometimes drawn from among the displaced. Both of these international jewels assumed that wild nature was best preserved in the absence of people. Even smaller preserves in various countries were established on land donated or purchased to maintain some natural feature or representation of biodiversity, subsequently constructing fences and regulating who could visit or use the preserve. In Europe some older preserves or parks were originally royal hunting grounds or noble estates, and so often reflected centuries of public exclusion.

Limitations of the “wild-without-people” model became clear over time (Baird and Leslie 2013). Most glaringly, the landscape management practices of Idigenous or agricultural or pastoral residents disappeared along with the people. Consequently, so did some of the species and biotic communities the reserves had been established to maintain. Prevention of fire and cessation of hunting were the most problematic alterations in conserved lands. Contemporary management attempts to correct these lapses (Rozzi 2012; Saviano et al. 2018; Ferretti-Gallon et al. 2021). Secondly, as society became aware of the questionable morality of indigenous exclusion (Taylor 2016), conservation strategies began to evolve. Conservation began to explore the human-land relationships that it had formerly ignored or purposefully removed. Much contemporary conservation strategy works with such human relationships to accomplish its goals (Rozzi et al. 2014). Ecology has benefitted from this relational perspective.

These four examples of relational disciplines or practices—political ecology, governance, urban design, and conservation—have helped urban ecology value relational approaches to science and practice. There are of course other relational disciplines (e.g., Ford and Airhihenbuwa 2010; Lave et al. 2018), but we hope these brief examples suffice.

## Evolving contexts for relational urban ecology

To this point, the paper has briefly introduced the multiple themes of relationality in urban ecology and explored key developments in the field itself as well as insights from some other disciplines and practices with which it interacts. This section presents an overview of the changing drivers of urban systems that the science must accommodate. This might be thought of as the changing ontology of the contemporary urban realm.

### Urgency of urban transformations

The urban components of the globe are changing rapidly. The familiar projection that 60% of the population will be classed as urban by 2050 is one indication of the urgency of understanding and working with urban change. In total, the proportion of the Earth’s land classified as officially urban is increasing. But urbanization—in the narrow sense of converting lands or populations from rural categories to urban status—is only one dimension of urban transformation. The changes *within* urban places are also widespread and impactful (Zhou et al. 2021a). Some older industrial cities experience thinning human populations, with concomitant abandonment of some infrastructure, reduced maintenance, or reduced revenues (Haase 2008; McGrath and Lei 2021). Urban transformation also includes expanding development on wildland-urban interfaces, say between towns or exurbs and forest or shrub lands (Alcasena et al. 2018; Radeloff et al. 2018). Leap-frog urban development also embeds settlements having essentially city structures in formerly rural or even wild landscapes.

The rates and complexity of these changes are remarkable (Westman et al. 2022). This means that past urban histories may give scant insight for urban living, planning, design, and management in future. Urban ecology thus shifts from explanation of historic development trajectories, and explanation of familiar spatial structures, which are biased toward the global north and industrial histories, to the work of imagining and examining future possibilities (Wu et al. 2014; Wagner et al. 2016; Zhu et al. 2019; Simone and Castán Broto 2022). This is an ultimate kind of relationality, one which must deal with novel combinations of drivers, responses, and interactions. The paper turns now to an overview of some of the coarse scale drivers of novelty and uncertainty in urban change, focusing on climatic drivers, technology, connectivity, migration, and demands for equity and justice.

#### Changes in driving climatic factors

Climate change is one of the most impactful drivers of urban change (Scheuer et al. 2017). With its interacting dimensions of planetary heating, sea level rise, and its alteration of seasonal patterns of temperature and rainfall, intensity of storms and intervals of calm, it is beginning to expose secondary, and increasingly disastrous effects for humanity (Field et al. 2012). The partial list includes increasingly intense and longer-lasting droughts, occasioned by changes in precipitation and extreme atmospheric and soil moisture deficit, more intense extreme heatwaves, increasing flood intensity and frequency including the effects of atmospheric rivers of moisture, increasing frequency and severity of wildfires, and more frequent hurricanes that track closer to coasts and reach higher latitudes (McPhillips et al. 2018; Covington and Pyne 2020; Camargo and Wing 2021).

#### Technological change

There are many ways in which technology is altering the structure and processes of urban places. A cogent example is the emergence of the “smart city.” Enabled by advances in Information and Communication Technologies (ICT) and big data analytics, smart cities use data collected via extensive networks of mobile devices and sensors installed by municipalities or organizations to guide decisions, actions, or flows of people, vehicles, and goods. This may be considered an adaptive and responsive coproduction of urban processes. Smart city technologies provide innovative solutions for multiple purposes, such as stewardship, monitoring, maintenance, coproduction, co-implementation, and co-management of urban ecological systems (Li and Nassauer 2021; Wellmann et al. 2023). Smart solutions have facilitated a transition from a socio-ecological view of urban systems, where technology is a subset of social systems, to a social-ecological-technological view that explicitly recognizes the multiple complex and dynamic interactions between social, ecological, and technological components (Kim and Son 2022; Chester et al. 2023).

When accessible to various societal groups, smart technologies can enhance the functionality of urban systems and their sub-systems such as “green” urban biological ecosystems. Regular monitoring is essential to ensure urban ecosystems' functional diversity and health (Branny et al. 2022). However, the rapid pace of environmental changes in the contemporary world makes monitoring based on traditional methods challenging and insufficient. Machine learning and satellite-based remote sensing technologies provide solutions for enhanced monitoring of ecological systems like urban forests (Locke et al. 2013; United States Conference of Mayors n.d.) and other forms of urban green infrastructure (Nitoslawski et al. 2021). Sensors are also used for real-time performance monitoring, allowing for a higher capacity to deal with unpredictable environmental shocks and better deliver ecosystem services through active interactions with stakeholders (Li and Nassauer 2021). For instance, real-time soil data monitoring to measure moisture levels helps lower plant/tree mortality by protecting them against droughts and heatwaves (Li and Nassauer 2021; Branny et al. 2022). Further, it facilitates automated and more efficient irrigation in water-stressed areas and contributes to urban resilience (Li and Nassauer 2021; Branny et al. 2022).

While smart technologies may increase automation, it is also possible to integrate social elements into such practices (e.g., through approaches based on citizen or participatory community science and data crowdsourcing), thereby providing more effective and efficient solutions for ecosystem resilience (Nitoslawski et al. 2021). Smartphone apps and web-based Geographical Information System (GIS) platforms offer tools that can foster resident awareness, improve the sense of place, streamline the connection between various stakeholders, and facilitate evidence-based decision making toward better managing urban ecosystems (Branny et al. 2022; Wellmann et al. 2023). For instance, the city of Melbourne is using an online digital platform to monitor the health of urban trees in collaboration with the public. The online tool allows residents to better understand the value of trees in the city and contribute to urban ecosystem management (Branny et al. 2022).

Some authors have cautioned that using AI and smart city approaches may reinforce existing inequalities and promote market-based exclusions in urban places, especially in the Global South (Bandauko and Nutifafa Arku 2023). This can be seen in urban contexts like India, where a quarter of the world’s urban South population resides, where smart city approaches have led to the de-prioritization of the provisioning services provided by urban commons to migrants and marginalized residents (such as grazing, fishing and fuelwood collection), leading to exclusion and alienation of the urban poor (Mundoli et al. 2017). AI and machine learning tools when applied using racialized approaches may exacerbate existing biases (Benjamin 2019). Similarly, use of community science or participant monitoring mobile device apps may under-represent marginalized neighborhoods or settlements (Ellis-Soto et al. 2022; Fernández-Álvarez and Gutiérrez Ladrón de Guevara 2022). Such bias may also appear in field-based methods (Gadsden et al. 2022). In a related vein, some authors emphasize the value and scalability of low-tech urbanism (Box 1). Equity issues relevant to AI and smart cities are further discussed in this Special Feature (Grove et al. 2024) and elsewhere (Galaz et al. 2021).

## Box 1. Low-tech urbanism

Low-tech is not a rejection of technology, but it aims at its fair and sufficient use, a balanced mix between simple and complex technologies, according to Philippe Bihouix’s vision (Bihouix 2020). The systemic, critical and ethical approach of low-tech is based on four principles in a logic of collective resilience: the discernment for the “sufficient” according to the uses, sustainable management of resources, conviviality (appropriation, accessibility of tools and knowledge…), the search for the right scale in the territories, organizations and socio-technical responses provided. Low-tech is in full coherence with principles that underlie the concept of bioregion, as an interpenetration between urban and rural spaces and sobriety (consumptions, flows, perpetuation of natural environments…).

To reduce pressure on land and to preserve biodiversity and ecosystem services is a question of searching for the most appropriate technologies -gray and green- and operating models, past, present or future: reducing the impact of building with the reuse, repair, material sorting; investing in under- or un-occupied spaces (wastelands, parking lots, vacant offices, roofs …). But planning for space is not enough. It is necessary to plan for time. For example, a single space can fulfill several functions through mixed use or fulfill different functions at different times. For example, desynchronization of road traffic can avoid the construction of new transport infrastructures.

**Objective "Net Zero Land Take"** To slow urban growth, and renature urban environments, the European Commission has proposed in the EU Environment Action Programme to 2020 to achieve ‘no net land take’ by 2050. In France, the ZAN objective (“Zéro artificialisation nette” for Net Zero Land Take)—part of the 2018 “Plan biodiversité”—is a turning point in strategies designed to slow urban sprawl as it places the emphasis on urban revitalization and densification. It also introduces a renaturing goal that involves “giving back to nature” an amount of land equivalent to that consumed by urban growth (Barra et al. 2022). A major advance, the ZAN objective is nevertheless subject to several criticisms: lack of numerical objectives and a clear time horizon, ambiguity of the term “net” which suggests the possibility of compensation rather than the elimination of impacts, and the definition of artificialization which can defeat the purpose of the project. In territories that are already highly urbanized and lacking of nature, an objective of zero gross artificialization and voluntary renaturation seems more coherent.

### Connectivity at global and regional scales

**Fig. 1:**
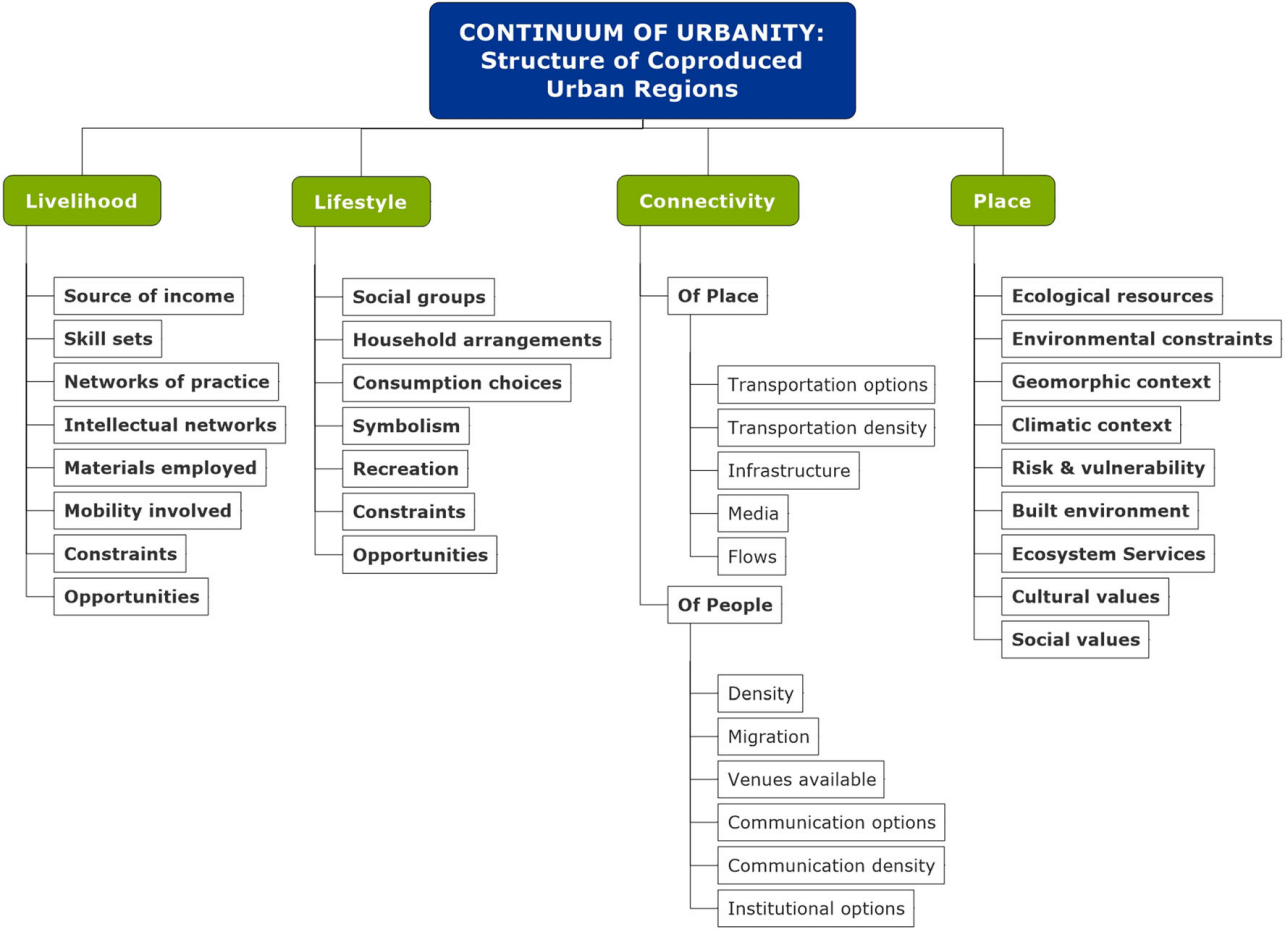
The four interacting components and lower level mechanisms of the continuum of urbanity, representing insights from Boone et al. (2014). Note: the scale can range between a region to the global

In the highly connected contemporary world, proximity is not the principal driver of urban life and economy (Angelo 2017). This concept suggests that rural–urban connectivities can be powerful relationships (Liu et al. 2007, 20; Liu et al. 2013; Brondizio et al. 2016; Delaroche et al. 2023). Important influences flow across urban regions and among urban areas across the globe (Plowright et al. 2011). Connectivity explains how urban structures and biophysical processes interact to generate urban transformations in specific locations as well as over larger regions (Fig. 1). It extends relationality over space (Elmqvist et al. 2021; Zhou et al. 2021b).

There are significant practical implications of connectivity. For example, it reminds urban ecology researchers, as well as policy makers, decision leaders, managers, and residents of urban regions, that not all important environmental actions can be circumscribed by jurisdictional boundaries. Nor can the processes of positive transformation in urban areas be successful if the familiar fragmented government and private institutional structures are not better linked or, indeed, integrated formally or informally (Delaroche et al. 2023). From both scientific and practical perspectives, recognizing the powerful role of connectivity helps overcome the narrow focus on bounded cities, towns, or villages, or even their seemingly discrete opposites in farmland, grazing lands, wild lands, or nature reserves (Brenner 2014a; Gandy 2014). Translating the dissolution of the human-nature divide emerging from contemporary conservation practice encourages the use of nature-based solutions in urban situations. See, for example, Box 2 on the Staten Island Bluebelt.

## Box 2. The Staten Island Bluebelt as a Nature-based Solution

**Fig. 2:**
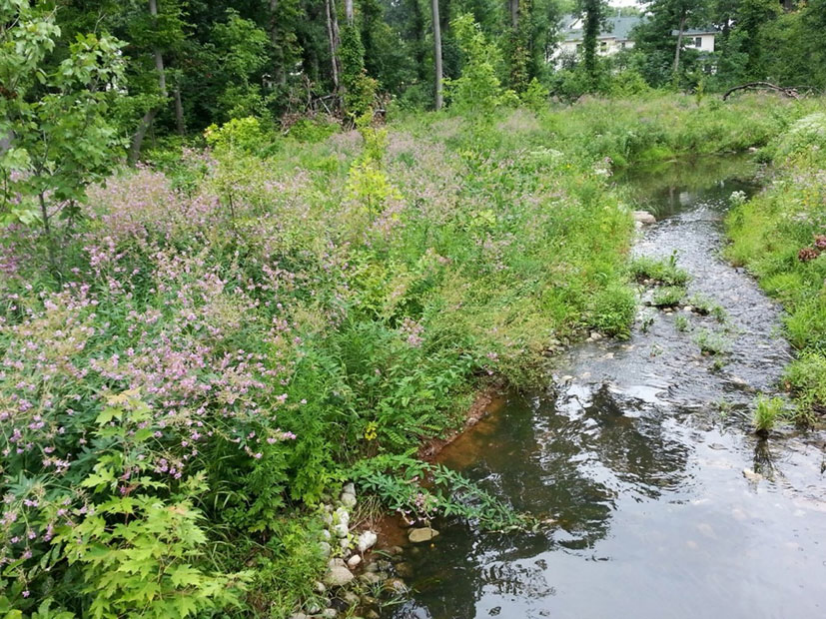
After creation of the Sweet Brook Bluebelt, Staten Island, New York. Photo by Matt Green. Attribution-NonCommercial-ShareAlike 2.0 Generic (CC BY-NC-SA 2.0)

The Staten Island Bluebelt is a system of wetlands created since the 1990s to provide ecosystem-based stormwater management in a rapidly developing borough of New York (NYC; Fig. 2). The Bluebelt program preserves natural drainage corridors such as streams, creeks, and ponds, and optimizes them to help control and filter stormwater from surrounding neighborhoods. The Bluebelt has become a widely replicated model for many cities as a way to provide multiple ecosystem services, including stormwater management, water quality improvement, wildlife habitat provisioning, environmental education, and increased property values.

**Fig. 3:**
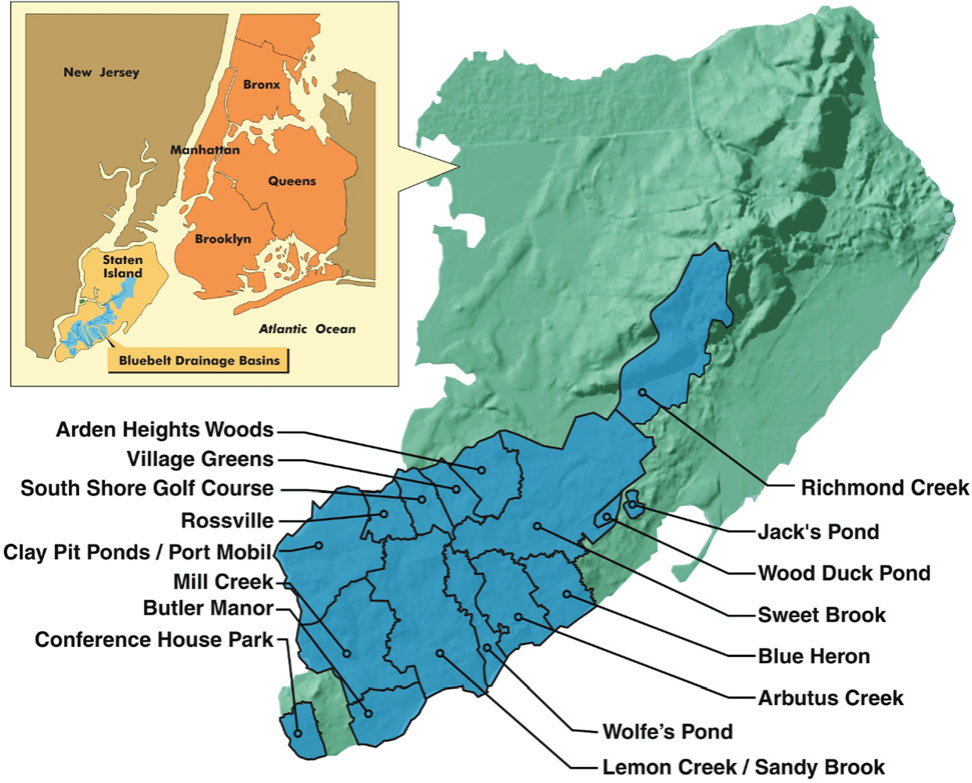
Location map of the Staten Island Bluebelt with detailed panel showing its 16 watersheds. *Source*: The Staten Island Bluebelt: A Case Study in Urban Stormwater Management by Dana Gumb, S. Mehrotra, and B. Henn, 2007

In 1990, the NYC Department of Environmental Protection developed the Bluebelt program and began constructing stormwater best management practices (BMP) along stream and wetland corridors to attenuate routine storm flow and improve water quality and flood flow (Fig. 3). The first phase of the Staten Island Bluebelt project was $48 million and estimated to generate capital cost savings of about $30 million following initial implementation. In 2020, the City announced that construction had begun on a $75 million enhancement of the Bluebelt program in the Mid-Island section of Staten Island.

The Bluebelt concept has two main goals: (1) to provide basic stormwater infrastructure and (2) to preserve the last remaining wetlands in Staten Island (Gumb et al. 2007). Since 1995, more than 70 sites have been developed under the Bluebelt program, all of which were justified by a cost–benefit analysis comparing Bluebelt development costs with those of a conventional piped stormwater storage system.

Bluebelt systems enhance and preserve natural areas while creating an efficient method for drainage of stormwater. Staten Island was directly in the path of the 2012 Hurricane Sandy, and the Bluebelt demonstrated its resilience and adaptability during the storm. Although the storm surge and intense precipitation from Sandy exceeded the treatment capacity of the Bluebelt, it returned to a functional condition soon after the storm passed. Bluebelts across Staten Island have proven to be an integral nature-based solution for managing stormwater and reducing flooding (NYC 2020).

### Human migration

One of the largest social drivers that attends climate change is human migration to escape such things as coastal inundation or consistent failures of rain fed growing seasons (Akinnagbe and Irohibe 2014; Vince 2022). These climate change motivations only reinforce the conspicuous differences in economic opportunity, political stability, access to education or medical care between city and country, or between the Global South and North (Akinnagbe and Irohibe 2014; Raleigh 2014) that have long driven migrations within and across national boundaries. Social movements of liberation can contribute to large migrations as well (Peet and Watts 2004). In a highly media-driven, globalized world, images of wealth, leisure, and choice that arrive from elsewhere can invite migration toward a better life (Clemens et al. 2014; Raleigh 2014; Grimm et al. 2016). In this world of image as opportunity, cities can be attractive not because they are, as many places in the Global North traditionally signified, sites of industrial or commercial production with their attendant opportunities, but can be loci where even marginalized persons can seek involvement in a wide arena of potentially productive activities (Simone 2010).

### Demands for equity and justice

The shift of urban ecology to a discipline and practice concerned with equity and justice is examined in detail in Grove et al.’s (2024) contribution to this special feature. However, because such concerns are widespread among people and organizations worldwide (Heynen et al. 2018), they must be included among the global motivations toward relational urban ecology. These concerns were powerfully expressed by Lefebvre in 1968 as the “right to the city” (King 2019). The persistence and salience of concerns for social and environmental justice have ranged from protests and action against contamination from waste dumps or industrial facilities (Bullard et al. 2008), to lack of access to employment in such movements as the Arab Spring, to protests and interventions focused on climate justice (Klinsky and Mavrogianni 2020), to the unexpectedly global reach of the Black Lives Matter movement (Hawthorne 2019), and widespread calls for the right to the city (Domaradzka 2018). The disruptions of COVID19 further highlighted the role of inequity and spatial segregation among oppressed populations (McPhearson et al. 2020). The breadth and nimbleness of urban mobilization (Domaradzka 2018; Mitlin 2018) have helped alert urban ecologists to better understand the causes and resilience of power, wealth, and information gaps and their relationship to environmental hazards and how they couple with social vulnerabilities (Chambers et al. 2022). These are clearly relational matters, called by historian of science, Gregg Mitman, “the ecology of injustice that structures urban life” (quoted in (Wolff 2007). Yet opportunities for online urban mobilization are undeniably shaped by factors such as access to the internet, which remains significantly lower in the Gobal South, contributing to structural imbalances between North and South cities (Nagendra et al. 2018). Concerns with justice are equally pressing on both sides of the Global North/South binary (Grabowski et al. 2022).

### Understanding rapid change demands relationality

The intersecting global urban drivers (Westman et al. 2022) just enumerated demand what can be seen as a relational approach (Solecki et al. 2013). Relationality has become so important for urban ecology because it is no longer possible to assume that the structures we observe are driven by the same conditions under which they were created or maintained in the past. Some authors acknowledge the novelty of combinations of drivers by employing the term, Anthropocene (Williams and Crutzen 2013; Elmqvist et al. 2021). This tactic suggests substantial discontinuity from the prevailing conditions of the Earth’s Holocene post-glacial history. Although the term is contested, it is clear that the contemporary urban world is vastly different than that of even a few decades ago (Taubenbock et al. 2014; McHale et al. 2015; Groffman et al. 2017). Whereas in the past, observing consistency in an urban entity through time, or documenting similarity in structure from place to place, may have been taken to imply similar levels and mixtures of drivers, that is certainly no longer the case. The relationships underlying the entities must be unpacked. Not only is this a new strategy for urban ecology, it is also the case for urbanism itself (Graham and Marvin 2001; Ellin 2013; Brenner and Schmid 2014). Change is now faster than empirical rules can keep up (Chester et al. 2023).

This brief exposition of individual factors that affect the relationality that urban ecology must address is only a start. In reality, relationships overlap, intersect, and influence one another. The emerging field of Recurrent Acute Disaster (Machlis et al. 2022) shows some of the complexity of how relational factors can interact in time and space, often in new or unexpected ways.

#### Recurrent acute disaster as an example of changing relationality

Recurrent acute disaster acknowledges that massive disturbances that can have catastrophic human, social, and economic impacts are generally increasing in intensity and frequency (Webster et al. 2005; Ting et al. 2019; Reichstein et al. 2021; Thompson et al. 2021; Machlis and Pickett 2022). The conceptual structure for dealing with such alteration of relationships in urban (and other) places works like this: First, biogeophysical disturbance events and the human disasters they trigger are more likely to occur in concert than in the past. For example, fires that are extinguished by rainstorms may be followed by floods fed by runoff from the denuded soil that is less absorptive of rainwater than when covered by vegetation and leaf litter. This phenomenon has been labeled compound or cascading disaster (Cutter 2018). Second, increased frequency means that individual events are likely to follow one another more closely through time. This is the phenomenon that has been labeled recurrent acute disaster (RAD), a conception that suggests new theory that exposes how a series of disasters are linked functionally through time (Machlis et al. 2022). As disasters occur more frequently, the legacies of prior events are all the more likely to persist long enough to influence how social-ecological-technological systems are affected by subsequent disasters (Ziervogel et al. 2017). In other words, legacies of earlier disasters can operate in ways that increase (or decrease) the vulnerability of a social-ecological system to subsequent disasters (Román et al. 2019). These new temporal and severity patterns upend the usual ways of planning for and responding to disasters. Official practice and media attention has usually treated disasters as individual, extraordinary events (Cutter 2018; Pescaroli and Alexander 2018). Now, however, it is important to develop policy and to refine public perception to think and act concerning disasters in a relational sense. They are not individual occurrences, but are more like assemblages of events related functionally through time (Machlis and Pickett 2022).

Second, vulnerability in such a series of recurrent acute disasters can accumulate. For example, vulnerability can be increased by social attributes such as emergency fatigue by the public, or exhaustion of responders, or depletion of stockpiled materiel in an earlier disaster. In the biophysical realm, soil stability, imperviousness, or wind firmness of trees can be altered so that a second disaster within a relatively short span of time wreaks greater damage than historical experience with individual disasters might suggest.

All disasters thus are both natural and social in nature (Hartman and Squires 2006; Grimm et al. 2017; Lugo 2018). The initial or primary motive force may emerge from the biophysical realm, but human disasters are coproduced by the interaction of a biophysical event with the social structure, composition, and artifacts, or technologies that determine what human impacts can result. Who and where are vulnerable are aspects of differential power relations (Lugo 2018; Parthasarathy 2018). Gibson et al. (2021) note that ecologists must be better trained to engage in the needed disaster research. Historical colonial legacies of divisive rule based on factors such as race, caste and gender often shape pre-existing vulnerabilities to disaster, especially in Global South cities, in ways that are far from well understood, with long-lasting legacy effects (Shackleton and Gwedla 2021). The next step takes this argument of urgency and novelty of urban change to a larger arena of coproduction as a key concept in a relational shift.

## Three kinds of coproduced relationality

The term “coproduction” can represent at least three different phenomena—coproduction of the city as place, of science, and of actionable knowledge (Rademacher et al. 2019; Pickett et al. 2021; Cadenasso et al. 2022). Each of these three kinds of coproduction reflects its own relationality.

### Coproduction of place or system

The first concept of coproduction details how urban places, habitats, and patches are hybridized by social and biogeophysical processes. Such hybrid places in the world can be labeled as social-ecological systems (Redman et al. 2004; Ostrom 2009; Lave et al. 2014; Brondizio et al. 2016; Folke et al. 2016) or as human ecosystems (Machlis et al. 1997; Naveh 2000), or as social-ecological-technological systems (SETS; Grimm et al. 2016). They are hybrids whether the focus is on the urban entities or their qualities (Marcotullio and Solecki 2013). Regardless of the label, they are coproduced by social and biophysical structures, processes, and interactions (see also Figs. 1 and 2 in Grove et al. 2024). Box 3 gives an example of the social-avian linkages in Phoenix AZ, US.

## Box 3. Ecology of the city: Social Processes and Bird Abundance and Diversity in Phoenix

The Central Arizona-Phoenix Long-Term Ecological Research Program (CAP LTER) has been studying urban ecology from a deeply interdisciplinary perspective for 25 years. An example of ecology *of* the city findings from this research combines data from the Phoenix Area Social Survey (PASS), conducted every five years, with data on bird community dynamics in the neighborhoods where residents are surveyed (Allen et al. 2019; Andrade et al. 2019; Brown et al. 2020; Wheeler et al. 2020). A decadal pattern of declining bird diversity throughout the Phoenix metro area (Banville et al. 2017; Allen et al. 2019) corresponded with a similar decline in diversity in the PASS neighborhoods. This social-ecological ecology *of* the city analysis revealed that resident satisfaction with the variety of birds in their neighborhoods paralleled this decline in bird diversity, and that residents of more bird-diverse neighborhoods were more satisfied with their local biodiversity (Warren et al. 2018; Wheeler et al. 2020).

### Coproduction of science

The second kind of coproduction shows that science is itself is a coproduct at the intersection of broader social, political, and cultural dynamics and assessments of the natural world (Longino 1990; Jasanoff 2004; Oreskes 2019; Wyborn et al. 2019; McKittrick 2020). As a social practice, scientific research is shaped by institutional arrangements, norms, and dialogs of testing the expectations from theories and models against the measurements of outcomes in the observable world (Latour 1993; Latour and Woolgar 2013; Orr et al. 2015).

### Coproduction of actionable knowledge

The fundamental coproduction of science and social order can explain the social context from which problems, issues, or ideas emerge as targets for actionable knowledge coproduction (Prescod-Weinstein 2021; Chambers et al. 2022). The various groups involved in coproduction of actionable knowledge likely have different values, knowledge systems, rewards, constraints, and time frames for action. Furthermore, they likely differ in their power (Ernstson 2013; Grabowski et al. 2023). Such richness is part of the social context of the interaction (Wyborn 2015; Muñoz-Erickson et al. 2017; Chambers et al. 2021). In addition to the “usual suspects” of scientists, government policy makers, agency managers, coproduction is seen to be just and equitable when Indigenous, racialized, local, traditional-knowledge holders, and lay participants are included and their voices and values recognized (Turnhout et al. 2020; Longo et al. 2021; Howarth et al. 2022; McGrath et al. 2023). Chambers and colleagues (Chambers et al. 2021) enumerate six techniques that are required to coproduce actionable knowledge: (1) researching solutions; (2) empowering voices; (3) brokering power; (4) reframing power; (5) navigating differences; and (6) reframing agency. With coproduction of knowledge as a process in which different knowledges weigh in, the relational understanding of people *with* nature and place become evident and productive for more positive connections with urban nature (Frantzeskaki and Kabisch 2016; Masterson et al. 2017; Frantzeskaki et al. 2021; Pickett et al. 2021). In other words, coproduction of knowledge is a highly relational activity. Not only is actionable knowledge coproduced, but to return to the fundamental fact that “nature” is itself coproduced (Cronon 1995), the actions of management, policy, and design interventions coproduce a new version or ontology of the system of interest (Cook et al. 2021; Chester et al. 2023). The city is as much a coproduct (Rademacher and Sivaramakrishnan 2017) as is nature in the contemporary world (Cadenasso et al. 2022).

## Motivations for coproduction of urban places

This section focuses on the coproduction of urban places as a key kind of relationality. Just as coproduction of knowledge involves diverse values, knowledge systems, motivations, constraints, and capacities, the same kind of diversity underwrites the human role in coproducing the city itself. Many of these drivers are well known in other disciplines, such as anthropology, political ecology, sociology, or economics, but bringing these perspectives and their insights to bear on coproduction in urban ecological science expands the understanding of urban places as complex social-ecological-technological systems (McPhearson et al. 2016). The conspicuous spatial heterogeneity of urban places reflects a wide range of social responses, adaptations, and tools, which individually and together interact with biophysical components of urban places (Folke 2006; Folke et al. 2016; Burch et al. 2017; Pickett et al. 2017). This section explores several important social phenomena that help determine the coproduction of social-ecological heterogeneity of cities, towns, suburbs, exurbs, and wildland–urban interfaces.

### Making a living

This aspect of urbanism refers to livelihood, or how people support themselves. Livelihoods range from subsistence to participation in markets. Livelihood can include formal and informal economic arrangements. Thus, making a living can rely on institutions that are long-lasting and formally or legally constituted. In contrast, it can take place in organizations that may have no legal charter or those which can operate outside legal pathways, or it can involve transitory, and ad hoc arrangements (Simone 2019). Such livelihoods can range from simple “off the books” work to organized cabals.

Livelihood activity can affect ecological characteristics and processes through several pathways. The direct harvesting of biotic resources alters composition and interactions in urban social-ecological systems, often informally managed common pool resources in the global South context (Mundoli et al. 2017). Notably, resource-based livelihoods can have either negative or positive effects on urban ecology through the dominance of either extraction or stewardship (Chapin et al. 2011a, b). The alterations of structure and process by a particular livelihood can cascade, and subsequently affect biodiversity in general, nutrient cycling, biological productivity and carbon sequestration, or decomposition of organic matter, for example (Chapin et al. 2011a, b; Petersen et al. 2012). Many aspects of ecosystem services in urban places may be affected by livelihood (Lambe et al. 2020). For example, heat mitigation or management of stormwater amount and quality may be constrained by the ecological effects of livelihood that add hard surfaces or reduce vegetation cover in settlements (Childers et al. 2019). Resources such as living organisms, lumber, packing materials, or soils that are brought into or through cities in the pursuit of livelihoods can introduce microbial disease agents or other organisms that achieve pest status when released from the limits of their ecosystems of origin (Lovett et al. 2016).

### Lifestyle

Lifestyle, which can be defined as the social signifiers that people express through their behaviors or surroundings, can affect ecology of the city (Grove 2014). Lifestyle effects can differ with religious identity, social or economic status, or racialized category. These and many other social identifiers affect the size of households, the sizes of structures occupied, residence in single- or multi-household dwellings, the modes of vehicular or other transportation employed (including what kind of car one drives if so resourced), whether a household is vegetarian or carnivorous, whether and how a property is vegetated and managed, the kinds and locations of environments chosen for recreation, and so on. Although it may be difficult to quantify or otherwise assess lifestyle factors, and there may be interactions among them and with livelihood, it is crucial for urban ecologists to recognize the rich body of relationships of social origin (Weiss 2000; Schneider et al. 2014; Moore 2015) that feed into biophysical structure and function across urban areas, reflecting coproduction. Furthermore, the social aspects of livelihood and lifestyle are heterogeneous and may shift spatially through time in an urban area. Quite disparate juxtapositions can appear in urban areas due to the conditions surrounding livelihood and lifestyle (Pieterse and Simone 2013), and buildings, grounds, and infrastructure may be informally or officially adapted for purposes different from their original design. In the global South, sacred and spiritual connections to elements of urban ecology, such as sacred trees and water deities, ant hills or bat roosting sites play an important role in influencing human-nature relations and impacting coproduction of ecosystems as sacred spaces. The role of lifestyle factors in influencing these informal connections is undeniable, but needs to be better understood (Gopal et al. 2019). In the Global North, parallel concerns are often addressed through Indigenous knowledge (Kimmerer 2013).

### “Making do”

This term encompasses the opportunistic social, economic, or habitation strategies enacted by persons or groups who must operate outside formal processes of markets, land tenure, housing construction, documentation, or municipal service provision (Simone and Pieterse 2017). Making do is a particular kind of coproduction of place conducted by refugees, migrants, or the oppressed who experience spatial or social exclusion. This term might seem at first to be just another label for the informal aspects of livelihood. However, the term is useful for indicating how common is the exclusion from reliable, gainful employment in the formal economy or lack of access to safe affordable housing around the world, and how common are creative or flexible social adaptations to these conditions (Shimamura et al. 2017; Simone 2019). The conditions entailing making do remind urban ecologists of the Gobal South/North disparities (Roberts and Parks 2006; Nagendra et al. 2018). Making do can also reflect isolation—or freedom—from formal municipal decision making. In many situations around the world, these kinds of exclusion are highly spatialized, as in the case of informal settlements of self-constructed dwellings. Not only are such places often underserved by municipal utilities and investments, but it may be illegal to occupy them, and so residents do not hold rights to property or representation in governance.

There are potentially many ecological implications of making do. First, places where people are allowed to settle or to practice resource use or business as they see fit, may be in environments neglected or abandoned by the powers-that-be due to perceived unproductivity, outright hazard, or environmental racism toward Black, Indigenous, or other marginalized communities (Waldron 2018; Anderson et al. 2020; see also Fig. 7 in Grove et al. 2024). Furthermore, the activities open to residents of such places may involve or produce hazardous substances that concentrate there or contaminate downstream and downwind locations. There are many other ecological questions that may be asked about the locations where people might live or gather in the course of making do: What kind of remnant, volunteer, or domestic biodiversity exists in such areas (Kremer et al. 2013)? How does that biodiversity compare with that in wealthier places or those better integrated into municipal decision making (Anderson et al. 2020)? What positive contributions might the biophysical ecosystems under the care of marginalized and underrepresented people make locally or in the adjacent neighborhoods, or in the whole city or region (McHale et al. 2018)? How can ecological research be useful to residents in informal settlements that are self-built, often on hazardous terrains, or for local resource-dependent communities (Gopal et al. 2015)? Such questions are a reminder that in situations where ecological science is practiced predominantly by individuals who are identified as racially or financially privileged, empirical work has often neglected marginalized neighborhoods (Gadsden et al. 2022).

The existence of “making do” places demands on transdisciplinary urban research practice. Simone and Pieterse (2017: xi) summarize the situation of “The largely makeshift complexion of many cities in Africa and Asia.” We believe this quotation to be relevant to marginalized places in North and South America and in Europe as well, indicating a dialogue between urban conditions in the Global South and North. Even in the USA, racialized segregation continues to exist, leading Hayes (2017) to refer to a “colony in a nation” that reflects the segregation of Black, Indigenous, and other persons of color. For example, Black communities in the U.S. have established a distinctive “interaction order” or mode of communication and mutual community support that attempts to protect them from the oppression of the white supremacist rank hierarchy exclusions to which they have for centuries suffered (Rawls and Duck 2020; Yacovone 2022). Indeed, the same kinds of creativity, flexibility, and communitarianism that Simone (Simone 2017, 2019; Simone and Castán Broto 2022) finds in Africa and elsewhere in the Global South are increasingly recognized as positive adaptations to oppression in the Global North (King 2018; Million 2018; Roane and Hosbey 2019).

The enormous transformations of the built environment and the enhanced possibilities of consumption that have marked even some of the most marginal of the world’s cities should not detract from acknowledging just how dependent the majority of the urban residents in these regions are upon constantly putting together some workable form of income and habitation (Pieterse 2011; Simone and Pieterse 2017). The makeshift character of much of what this majority does is quite literally ‘make + shift’” (Simone and Pieterse 2017; xi). Furthermore, scholars must also engage in this dynamic makeshift. “Thus, we locate our research and propositions within a *relational epistemological force field*…” (Simone 2004; xii, emphasis added). These insights suggest that the urban situations where urban ecologists work in both the Global South and North, and their approaches to research are both profoundly relational (Ellis-Soto et al. 2022; Gadsden et al. 2022).

### World making

Social scientists, philosophers, and activists speak of “world making” as a social process (Blaser and de la Cadena 2018; Nishime and Hester Williams 2018). This idea suggests that rather than there being a single, universal, shared world of a given city, there are in practice many social worlds in a city. In fact the major processes already outlined in this section are aspects of world making. The many differences in livelihood, lifestyle, and making do combine to generate a number of distinctive worlds inhabited, valued, and perhaps set in opposition to the worlds of others. Speaking in terms of “worlds” is different than speaking of world *views*, since that latter idea assumes that the city is a single *world* equally available and experienced similarly by all (de la Cadena and Blaser 2018). The different mental maps and knowledge systems that residents of various neighborhoods or social positions use in order to know and to orient their daily lives in the urban space are examples of the distinct worlds that can exist within even within a single city (Lynch 1960; Muñoz-Erickson 2014). Even when people’s daily orbits intersect, they may have different conceptions of their individual worlds. For example, even when workers and managers lived on the same block in the nineteenth century industrial heyday of Baltimore, their worlds orbited around the main streets of the block in the case of bosses, and the narrow back alleys in the case of the manual laborers and domestic workers (Hayward 2008). The plural term ecologies is rarely used in science, but may be appropriate for acknowledging the variety of social-ecological worlds (Pickett et al. 2022) that the different classes or racialized groups in cities experience. How these different social worlds and epistemic cultures influence ecological structures and processes is a major issue for research (Muñoz-Erickson 2014; Simone 2017; Locke et al. 2021). Therefore, a relational understanding of urban ecologies can facilitate the multiplicity of social worlds and urban meanings and so enrich urban lifestyles and their involvement in coproduction.

Urban places contain more subtle examples of world making as well. Some are gendered, as in the case of some U.S. post World War II white suburban communities where wives, who stayed at home to care for their households, experienced different worlds than their commuting husbands (England 1993), and how the gendered experiences of white housewives contrast to the long history of Black women working both in and outside the home (Palmer 1983; Jones and Shorter-Gooden 2009). Similarly, cultural worlds of LGTBQ persons can spatially overlap in cities but retain distinct circuits and activities from those who identify as heterosexual (Roane 2020). While such social and perceptual heterogeneity may seem distant from the concerns of ecology, it is just such complex worlds that can influence how people perceive, know, and manage the natural components of their surroundings (Delia et al. 2017; Hoyle et al. 2019; Kim and Son 2022). Indeed, inhabitants of some social worlds may hardly see nature at all in the city, while others are attuned to the activities and poetics of their other-than-human neighbors. It can be crucial for the work and impact of ecologists in urban systems to recognize how different human worlds see, know, or deal with “nature” in the city. Many Indigenous or oppressed caste groups in Indian cities may relate to nature in ways similar to kinship relations, expressed through song, sacrificial offerings and sacred worship for instance (Sen and Nagendra 2023). The recognition of the importance of different social worlds fills out our understanding of the coproduction of nature in the city. In fact, different social worlds can coproduce different aspects or states of ecological structures and processes in cities. These are material outcomes of different worlds (sensu Blaser and de la Cadena 2018), and ecologists must recognize this.

## The continuum of urbanity as a tool for unpacking the themes

This paper has already mentioned at several junctures the increasing impact of urban connectivity on various scales. This section now focuses on connectivity as part of a synthetic framework that helps synthesize different aspects of relationality in urban systems. Connectivity challenges much received urban theory and practice that assumes proximity to be a principal driver in urban systems (Huriot and Thisse 2000; Pickles 2006). Classically, cities generate value by serving as economic centers, bringing diverse people together to spark creativity, permit division of labor, or concentrate resources. These classical attributes were in many societies literally walled off from adjacent pastoral, agricultural, or wild lands or from populations perceived as hostile. Over the long term, but especially in the current globalized era, walls have disappeared and urban boundaries have become remarkably porous (McHale et al. 2015). The relational shift relevant here is that urban places are becoming more connected with both distant urban places, but also with the nearer territories experiencing different degrees of urbanization. The longer distance exchanges and influences are labeled teleconnections (Seto et al. 2012; Liu et al. 2013), and involve not only other urban areas, but rural and wild systems as well (Brondizio et al. 2016). Examples of teleconnection include clearing Amazonian rain forest lands to both grow soybeans and to raise animals destined to supply the growing number of middle class tables in China, or the fast food "joints” of the U.S. Further urban connectivities appear in the mining of trace minerals in some African countries to build cell phones ultimately headed to Europe, or the mining of lithium in Chile’s high desert to build the world’s electric vehicle batteries, both of which exemplify “unequal ecological exchange” (Jorgensen 2009), a species of power. Similarly the movement of CORONA19 virus across the entire settled world emphasizes connectivity. Not only organisms and material resources flow via teleconnections, but ideas, finances, and fashions flow as well. The very idea of the national park as a conservation reserve where human activities are intentionally minimized, was exported around the world from its elite, male-oriented recreational lifestyle roots in North America or Europe (Taylor 2016) to Africa, Asia, Australia, and South America. The extirpation and dispossession of Indigenous peoples and their lifeways often accompanied the establishment of national parks around the world. In the realm of lifestyle, Reggae and sneakers are now worldwide phenomena, far from their places of birth. Remittance income flows from migrants working in distant service or industrial economies to elders and children who have remained in the home country or in places that retain seemingly rural structures. Such migrations may be within a country as well as international. Henri Lefebvre (2003, originally in 1970), alerted urbanists to the pervasiveness and growth of such connectivity driven by urban ideas and resources.

A group of urbanists, planners, geographers, and ecologists jointly conceived a transdisciplinary “continuum of urbanity” (Boone et al. 2014) that helps operationalize Lefebvre’s insight in a transdisciplinary biophysical and social realm. The continuum assumes that in the contemporary world, virtually all places reflect some combination of wild or rural structures and processes along with urban structures and processes. The continuum identifies connectivity as the main determinant of the mixture of urban and rural characteristics of individual places. But the human and natural contributors are embedded in the livelihoods practiced in those places, the lifestyles expressed there, and the biophysical and constructed aspects of the places. Work exploring notions of the urban in the Global South recognize the role of history, economy, and culture in positioning settlements on this continuum (McHale et al. 2013). As mentioned earlier, both livelihood and lifestyle are capable of influencing the environment through patterns of consumption, waste, or aesthetics. Connectivity brings new resources, new expectations, new capital, or new populations—temporary or permanent—to or from places well beyond the city (e.g., Brondizio et al. 2016). As a theoretical structure, the continuum of urbanity posits many specific mechanisms by which livelihood, lifestyle, connectivity, and social-ecological system structure can interact (Fig. 1). The spatial complexity, temporal dynamics and feedbacks, and interaction of the processes that the continuum specifies illustrate a major set of relational shifts affecting the science of urban ecology and the increasingly region-wide, lived urban worlds.

## Eight themes of relational urban ecology

Eight themes emerge from the explorations in this paper. Some are parallel to long standing developments in other fields active in urban research and practice. Some reflect roots in ecological science. But all combine in advancing urban ecology in particular and urban science in general (Solecki et al. 2013). Because they help link the diverse relationships necessary to understand urban places, the themes help to position urban ecology to better contribute to positive urban futures for people and for the environment.

1. Urban is no longer congruent with “the city.” This tradition can be exemplified by Lefebvre (Lefebvre 2003). Following suit, recent authors have critiqued both the methodological and theoretical anchoring of urban studies in the city (Brenner and Schmid 2014). This leads us to question urban ecology as a study of cities as discrete, well-bounded entities. At the least, this perspective indicates to urban ecologists that even when focus might legitimately be on particular cities, they have diffuse boundaries and are remarkably open (Gandy 2014; McHale et al. 2015).
2. There has been a tradition of seeing cities as monumental and permanent (Scully 1993). The figure-ground representations of cities are a part of this tradition. But urbanists are increasingly recognizing that there are “generational” turnovers in urban places (Shane 2005; McGrath 2016), based on such things as obsolescence or degradation of buildings and infrastructure, changes in economic drivers and opportunities, growth and senescence of urban plantings, new technologies, and social and demographic shifts in density and use. Depending on local and regional particulars, each of these kinds of change can impact urban places in new and unfamiliar ways. In this context of urban dynamics and revitalization, opportunities arise for coproduction of new urban ecologies shared by people having different worldviews. Such new ecologies can create places in which people can experience new connections with nature. Understanding the mutability of cities also has an urgent application in the field of disaster ecology. Given that urban infrastructure, emergency preparedness, and disaster response are keyed to past experience, adaptation to novel and still changing environmental disturbance and hazard regimes is an urgent issue (Machlis and Pickett 2022). Not all urban mutability is intentional.
3. Themes one and two have recognized two kinds of novelty in how people must think about and manage cities, including for restoration (Larson et al. 2013) or future visioning (Cook et al. 2021). Together, those themes point to the core message of this paper: The study and operation of urban systems, in cities and beyond, must increasingly focus on relationships. This insight of course parallels the emphasis of critiques of modernism that suggest focusing on relationships first, rather than emergent structures (Latour 1993; Donati 2021). This insight is also familiar in complexity theory (Allen and Starr 2017). Furthermore, the idea should be familiar to ecologists, given that modern definitions of the discipline center relationships: Ecology is *the study of the processes influencing the distribution and abundance of organisms, the interactions among organisms, and the interactions between organisms and the transformation and flux of energy, matter, and information* (Weathers et al. 2021). Note that the term “processes” in the definition could be replaced by “relationships.” Urban ecology is beginning to take interactions as central to its mission.
4. Relationships extend through time and over space. Thus, urban systems must be understood as embedded in extensive spatial networks of environmental relationships and social relationships. We take each of these two complementary realms in their most comprehensive senses: Environment includes soils, waters, energy, and non-human organisms from microbes to megafauna (Pickett and Grove 2009). The social realm encompasses economy (Daily and Ruckelshaus 2022), unequal power relations (Turnhout et al. 2020), modes of governance, (Delaroche et al. 2023) institutions (Ostrom 2012), technology (Grimm et al. 2016), and flows of ideas (Pellow 2016), among others (Burch et al. 2017). The spatial extent of urban processes is highlighted by the continuum of urbanity (Boone et al. 2014), described above, as well as other spatial concepts such as megacity, urban region, metacity (McGrath and Shane 2012), urban agglomeration, planetary city (Brenner and Schmid 2014), and telecoupling (Brondizio et al. 2016) to name a few.
5. Importantly, relationships have a temporal dimension, so that lags and legacies are often crucial in tracking how relationships unfold, or what their consequences are. This differs from the attention of ecology in its early years on primarily instantaneous, universal relationships that might be represented by invariant laws like “succession to climax” (Pickett et al. 2007; Simberloff 2014). That view may be useful in some idealized situations where path dependency or contingency in how current conditions have come to be can be ignored, but there are many “non-equilibrium” situations where past conditions are influential into the present or future. Historical contingency is crucial where large, long-lived organisms play a role in urban relationships—for example trees in urban ecosystems (Anderson et al. 2023), or where the past policies such as segregation (Grove et al. 2018; Pickett et al. 2023), or highway routing (Reft et al. 2023) have lasting social-ecological signatures. Contemporary associations between social and biophysical features of cities are not always adequate to understand the operative relationships. Urban ecology will be most amenable to positive futures when it is not bound by the familiar and persistent Western separation of people from nature. Again, critics of modernism (Donati 2021) have explored this intellectual territory, as have critics of conservation based on exclusion of Indigenous people (Chapin 2004; Taylor 2016), and recent research has similarly explored these issues in cities of the global South (e.g., Sen and Nagendra 2023).
6. Multiple conceptions of coproduction can help urban ecology prepare for positive futures: first coproduction of urban structure by both society and nature (Cadenasso et al. 2007; Lachmund 2013; Rademacher et al. 2019), and second, as the process of solving problems through dialog between researchers, practitioners, and communities. Indeed, some scholars suggest that it is most productive for various experts and residents to “inhabit” a problem together as a key to co-designing solutions (Mitchell et al. 2015). That is, the field can contribute to improving urban places neither by relying *only* on so-called natural phenomena, nor *only* on socio-economic-political-technological perspectives (Frantzeskaki et al. 2019). In addition, as noted above, the interactions important to urban systems spread widely, often anchored in or impinging on places usually called “natural” that exist at a distance from dense urban nodes. The relational shift suggests that the dichotomy between natural and urban be usefully replaced by ideas of hybridity and coproduction of place.
7. Urban ecology is not a universalizing discipline. Urbanism theory and practice has in the past theorized urbanization as a developing “growth machine” (Kirkpatrick and Smith 2011). However, contemporary urban ecology does not assume that all urban places go through the same trajectory of “development.” Indeed, the contemporary urban world is quite diverse in form, process, and transformation (McHale et al. 2015). Each urban place must be taken to some extent on its own terms, in its own context, and in light of its own history. Emerging understandings around the complexities of urbanization, informed as it is by, for example history, culture, and economy, affirm no single trajectory to ‘urbanised’, a point to be mindful of when considering outcomes across the Global North and Global South, as well as within these regions. Of course, there are some things that most urban places share. For example, the reliance on resources from beyond their borders is a ubiquitous urban principle. There remains a need for an effective taxonomy of urban places so as to facilitate comparison, and to identify generalizations within specific scopes.
8. The final principle is to acknowledge that the human populations that depend on and reside in cities and urban regions differ greatly in access to perceived status, power, and resources (Boone 2008; Boone and Fragkias 2012; Anguelovski and Connolly 2021). This differentiation includes those whose addresses are in city, suburb, or rural jurisdictions. Urban ecology, in part a descendent of evolutionary biology, has come to recognize that racialized, class, and other categories of social status are not inherently biological (Graves 2003; Heynen 2016; National Academies of Sciences 2023). This is in spite of discredited work by earlier generations of biologists that supported racism and eugenics, and were used to justify colonialism, displacement, genocide, and slavery (Baker 2021). Contemporary urban ecology has been employed to identify and help overcome some of the biotic legacies of past and ongoing social inequities (Grove et al. 2018; Pickett et al. 2023). It has clarified such things as the factors that have generated contrasts in access to green space, parks, and tree canopy cover, or the exposure to hazardous sites, or exposure to hazards triggered by atmospheric or hydrologic disturbances, or pandemics (Sharifi 2022). As a consequence of these sorts of differences, and their deep histories in and around cities, urban ecology must take social heterogeneity and equity into account (Schell et al. 2020). In this way, urban ecology can generate more comprehensive data on urbanized places, and be of service to communities and constituencies that are usually disempowered.

## From understanding to action

The relational turn in urban ecology, enumerated in the eight themes above, can help improve how the field contributes positively to the inevitable increasingly urban future. The vast majority of humans born in future decades will live in urban places (United Nations Department of Economic and Social Affairs Population Division 2019). However, fully half of those urban places, whether cities, suburbs, exurbs, or urbanized rural and wild places, have yet to be constructed (see Frantzeskaki et al. 2024; Frantzeskaki et al. 2024). This provides tremendous opportunities for transformative action fueled by the relational thinking. (See Box 4 for an example about how shared knowledge of change in Kano City, Nigeria informs contemporary environmental action toward greening.)

## Box 4. From knowledge to action in Kano City, Nigeria

Kano city, in Northern Nigeria represents the story of the experiences of an African city’s society and ecology—the good, the bad, and the ugly. Old films, photographs, maps sketches, and travelers’ diaries illustrate the appealing and amazing nature of the city’s landscapes in the 19th and early twentieth centuries. The city’s landscape was shaped by an age-long socio-ecological system that underpins the city’s resilience dynamics over space and time. There are inspiring statements like this: *[T]he whole scenery of the town in its great variety of clay houses, huts, sheds, greens open places affording pasture for oxen, horses, camels, donkeys and goats, in motley confusion, deep hollows containing ponds overgrown with the water plant, the* Pistia stratiotes*, various and most beautiful specimens of the vegetable kingdom, particularly the fine symmetric gonda or papaya, the slender date-palm... silk cotton-tree... the people in all varieties of costume formed almost animated and exciting scene* (Barth 1857: pp. 492–493). After some 100 years another expression came thus: *“And a variety of bird and insect life flits about gaily, while in the inside the rich, intensively cultivated farms of the Kano countryside, scattered with large shady trees and pyramid-shaped piles of dawa (guinea corn) stalks.* (Moody, 1969 p.32).

Conversely, satellite imageries illustrate the extent of massive degeneration essentially from the early 1990s and worse scenes in the 2000s. Massive urban population increase and densification of urban landscapes stamped out the heritage urban ecosystems of Kano. The period 1970s/1980s was the gestation period for hatching local urban planners and bureaucrats. These experts and bureaucrats lacked training on and appreciation of the African city’s ecology. Oftentimes, their conceptualization of the urban discards the essential ecosystems.

Beyond despair, there are new hopes, actors, opportunities and possibilities for recreating the lost trees at least. A living lab stationed at Bayero University Kano has reintroduced some indigenous trees into olden neighborhoods named after some indigenous trees. Such action has inspired new actors, especially youth groups to engage in tree planting activities within their neighborhoods. These little actions will grow into big results for the city in the coming years.

Urban ecology’s transformative, relational power emerges not only from the dynamic urban trajectory, but from the discipline’s position at the boundaries of so many other urban relational fields of study (Solecki et al. 2013; McPhearson et al. 2016), its growing understanding of the three dimensions of coproduction, as well as its potential significance to those who struggle for better, fairer participation in their urban worlds (Grove et al. 2024). Importantly, the transformative power of the relational shift also emerges from acknowledging and reflecting on the implication of positionality of urban ecology and urban ecologists as both observers and actors within social-ecological-technological systems. It is crucial to avoid linearity, instrumentalism of knowledge, and faulty power sharing in attempts to co-create knowledge (Chambers et al. 2021; Sokolova 2023). We believe that the relational shift in urban ecology responds to the momentous global changes underway, and exploits the growing sophistication within the field itself. The dialog between these external and internal changes suggests that urban ecology is in fact well poised to meet what was so long ago identified by one of ecology’s founders (Clements 1935) but which remains salient today: How to meet change with change.
